# Donor age as an independent predictor of inferior outcomes after haploidentical hematopoietic cell transplantation in acute myeloid leukemia. Study conducted on behalf of GETH-TC

**DOI:** 10.3389/fimmu.2025.1700463

**Published:** 2025-11-28

**Authors:** Daniel Munárriz, Estefanía Pérez-López, Carlos Martín Rodríguez, Marta Luque, Albert Esquirol, Carmen Martín Calvo, Clara Aparicio, Felipe Peña-Muñóz, Inmaculada Heras Fernando, Itziar Oiartzabal Ormtegi, Adolfo Jesús Sáez Marín, Sara Fernández-Luis, Juan José Domínguez-García, Sara Villar Fernández, Jose Luis López-Lorenzo, Cynthia Acosta Fleitas, Ana Pilar González-Rodriguez, Lucía García, Tamara Torrado, Silvia Filaferro, Pascual Balsalobre, María Jesús Pascual Cascon, Montserrat Rovira, María Queralt Salas

**Affiliations:** 1 Hospital Clínic de Barcelona, Barcelona, Spain; 2 Complejo Asistencial Universitario de Salamanca, IBSAL, Salamanca, Spain; 3 Hospital Regional Universitario de Málaga, Málaga, Spain; 4 Hospital de la Santa Creu i Sant Pau, Barcelona, Spain; 5 Hospital Reina Sofía de Córdoba, Córdoba, Spain; 6 Hospital Duran i Reynals, Institut Català d’Oncologia, Barcelona, Spain; 7 Hospital General Universitario Morales Meseguer, Murcia, Spain; 8 Hospital Universitario Donostia, Donostia, Spain; 9 Hospital Universitario 12 de Octubre, Madrid, Spain; 10 Hopsital Universitario Marqués de Valdecilla, Santander, Spain; 11 Clínica Universidad de Navarra, Pamplona, Spain; 12 Fundación Jiménez Díaz, Madrid, Spain; 13 Hospital Universitario de Gran Canaria Doctor Negrín, Gran Canaria, Las palmas de Gran Canaria, Spain; 14 Hospital Universitario Central de Asturias, Oviedo, Spain; 15 Hospital Universitatio Son Espases, Palma de Mallorca, Spain; 16 Hospital Universitario de A Coruña, A Coruña, Spain; 17 Grupo Español de Trasplante de Progenitores Hematopoyéticos y Terapia Celular, Madrid, Spain

**Keywords:** donor age, allo-HCT, haplo-HCT, PTCY, AML

## Abstract

This study evaluated the impact of donor age on clinical outcomes in 274 patients with acute myeloid leukemia (AML) haplo-HCT using PTCY-based prophylaxis. Median patient age was 53 years, with 42.6% classified as high-risk AML. The median donor age of 38 years; 31% were under 30. An optimal donor age cut-off of 30 years was identified through ROC analysis. Patients receiving grafts from younger donors (<30 years) showed lower rates of aGVHD grade II–IV (3.0% vs. 19.9%, p < 0.001) and grade III–IV (1.5% vs. 10.2%, p = 0.034), with no differences in cGVHD or relapse rates. Overall survival (OS) was higher in the younger donor group (2-year: 80.6% vs. 64.3%, p = 0.011), along by lower non-relapse mortality (NRM) (2-year: 11.1% vs. 23.2%, p = 0.031). Multivariate analysis confirmed donor age ≥30 years as an independent adverse factor for OS (HR: 1.88, p = 0.019) and NRM (HR: 2.06, p = 0.049), along with older recipient age, higher HCT-CI score, and high-risk AML. These findings suggest that younger donor age contributes to improved survival, primarily through reduced NRM and aGVHD, supporting prioritization of younger donors when multiple haploidentical options are available to optimize transplant outcomes.

## Introduction

Allogeneic hematopoietic cell transplantation (allo-HCT) is a potentially curative strategy for patients with acute myeloid leukemia (AML). The effectiveness of allo-HCT in AML patients resides in the anti-leukemia cytotoxicity gained from the conditioning regimen and the allogeneic donor-cell graft-versus-leukemia (GVL) effect. Furthermore, due to its efficacy in achieving long-term disease control, the indication for allo-HCT in AML patients has been expanding (1). This is largely due to advances in induction therapies, the availability of clinical trials, and the growing number of older patients now eligible for curative-intent therapies.

Post-transplant cyclophosphamide (PTCy)-based prophylaxis has become a widely adopted strategy for preventing graft-versus-host disease (GVHD) in haploidentical allo-HCT (haplo- HCT). The introduction of PTCy has played a transformative role in expanding access to allo- HCT by making haplo-HCT a viable and safer alternative in the absence of matched donors (2). However, donor selection in the haplo-HCT setting remains complex, and optimal criteria are still under investigation (3). Among various donor characteristics, donor age has emerged as a significant factor impacting transplant outcomes. Younger donors are generally associated with lower rates of chronic GVHD and better overall survival, making age a key consideration in donor selection. As haplo-HCT increases access to transplant for patients who previously lacked suitable donors, understanding the role of donor age and other clinical variables becomes increasingly important to refine donor choice and improve patient outcomes (4).

The present study aims to investigate the impact of donor age in patients with AML undergoing haplo-HCT. Specifically, it seeks to identify how donor age affects clinical outcomes, transplant-related toxicity, GVHD incidence, and disease relapse. Ultimately, the goal is to generate evidence that can support donor selection in the haploidentical setting, especially in cases where more than one potential donor is available and age becomes a distinguishing factor.

## Methods

### Study design and patient selection

This is a retrospective and multicenter, registry-based study sponsored by the *Grupo Español de Trasplante Hematopoyético y Terapia Celular* (GETH-TC), a non-profit scientific society comprising members from all institutions performing HCT in Spain and Portugal. All institutions members of the GETH-TC were invited to participate, and16 transplant centers affiliated with GETH-TC participated in the study.

The study sample included 274 adult AML patients who underwent their first peripheral blood (PB) haplo-HCT at the participant institutions between 2011 and 2022. Retrospective data were collected through retrospective chart review and updated in December 2024. Data collection and management were performed using REDCap electronic data capture tools hosted by GETH-TC. The study was approved by the Ethics Committee of Hospital Clínic de Barcelona and adhered to the ethical standards of the Declaration of Helsinki. No external funding was received.

### Donor election practices across institutions, allo-HCT information and main definitions

Each transplant center followed their internal donor selection methodology during the study period. High resolution DNA typing for HLA-A, -B, -C, -DRB1, and -DQB1 was conducted in recipients and donors. All patients underwent allo-HCT from haploidentical first-degree relative donors. An HLA- matched sibling donor (MSD) followed by a 10/10 HLA-matched unrelated donor (MUD) were generally preferred upfront, and haploidentical donors were considered in the absence of a HLA-matched donors. Donor age was considered the main explanatory variable for the study conduction. Unfortunately, data related to donor/recipient CMV serostatus and ABO compatibility were not recorded. Induction therapies, eligibility criteria for allo-HCT, donor selection, conditioning regimens, and GVHD prophylaxis followed the standard protocols of each participating institution. Conditioning regimen intensity was tailored to each patient’s age and comorbidities. Acute and chronic GVHD (aGVHD and cGVHD) were graded according to established criteria. Complete remission (CR) and disease relapse were determined by the treating physician and recorded in the database.

### Statistical analysis

The primary objective of this study was to assess the impact of donor age on outcomes, including overall survival (OS) and non-relapse mortality (NRM). Donor age was analyzed as a continuous variable and as a dichotomized variable, using a 30-year cut-off determined by receiver operating characteristic (ROC) curve analysis. Additional relevant outcomes included the cumulative incidence of relapse (CIR), cumulative incidence of GVHD, and GVHD-free/relapse-free survival (GRFS).

Continuous variables were assessed using Student’s T-test or appropriate non-parametric methods. Categorical variables were summarized as frequencies and percentages and compared using Fisher’s exact test. OS was estimated using the Kaplan–Meier method, while NRM was estimated using the cumulative incidence function. The cumulative incidence of GVHD was calculated accounting for death and relapse as competing events. CIR and other post-transplant complications were analyzed death as competing event. GRFS was defined as the time from transplantation to the first occurrence of grade III–IV a GVHD, moderate/severe cGVHD, disease relapse, or death from any cause. Patients alive and free from these events were censored at last follow-up. The impact of donor age on OS and NRM was assessed with univariate and multivariable regression analyses (UVA and MVA). Variables found significant in the univariate analyses, or deemed clinically relevant, were included in the MVA. The proportionality assumption was evaluated using Schoenfeld test, with no violation detected.

All p-values were two-sided, with statistical significance defined as p < 0.05. Analyses were performed using RStudio (version 2024.04.4), EZR, and GraphPad Prism 10.

## Results

### Patient characteristics and main outcomes

Baseline characteristics of patients and donors are summarized in **Table 1**. Among the 274 patients included, the median age was 53 years (range, 17–74), with 62 (22.6%) patients over 64 years. A total of 123 (45%) patients were female, and 99 (36.8%) had a Hematopoietic Cell Transplantation Comorbidity Index (HCT-CI) > 2. According to the European LeukemiaNet (ELN) classification (2017 or 2022 depending on the date of diagnosis), 116 (42.6%) patients were classified as high-risk.

**Table 1:** Baseline characteristics of the study cohort and according to donor age.

Variables	All patients N=274	Donor age < 30 years N=85	Donor age ≥ 30 years N=189	P value
Age, median (range)(years) Older ≥65 years, total (%)	53 (17-74) 62 (22.6)	53 (17-69) 10 (11.8)	53 (17-74) 52 (27.5)	0.916 **0.004**
Sex, total (%) Female	123 (44.9)	35 (41.2)	88 (46.6)	0.433
ELN^1^ Classification, total (%) I-II III	156 (57.4) 116 (42.6)	52 (61.2) 33 (38.8)	104 (55.6) 83 (48.7)	0.429
Disease status prior allo-HCT^2^, total (%) Complete Remission 1 Complete Remission 2 or more	220 (80.3) 54 (19.7)	68 (80) 17 (20)	163 (83.2) 37 (19.6)	1
HCT-CI^3^ ≥3, total (%)	99 (36.8)	29 (34.1)	70 (38)	0.588
Intensity, total (%) Myeloablative (MAC) Reduced intensity (RIC)	145 (52.9) 129 (47.1)	48 (56.5) 37 (43.5)	97 (51.3) 92 (48.7)	0.436
Transplantation period After 2020	115 (42.1)	35 (41.2)	80 (42.3)	0.91
GVHD^4^ Prophylaxis PTCY^5^-CNI^6^ PTCY-CNI MMF^7^ PTCY Sir^8^ PTCY-Sir-MMF	27 (10.1) 238 (89.1) 1 (0.4) 1 (0.4)	8 (9.6) 75 (90.4) 0 (0) 0 (0)	19 (10.3) 163 (88.6) 1 (0.5) 1 (0.5)	1
Post-HCT Follow-up, median (IQR)(days)	35.5 (7.2-52.7)	39.1 (15.6-60)	34 (6.3-49.1)	0.195
Donor Information Age, median (range)(years) Female to Male Stem Cell Products, total (%) Cryopreservation, total (%)	38 (13-75) 45 (16.4) 49 (24.1)	24 (13-30) 15 (17.6) 18 (28.1)	42 (31-75) 30 (15.9) 31 (22.1)	**<0.001** 0.726 0.38

^1^ELN, European LeukemiaNet risk classification. ^2^Allo-HCT, allogenic Hematopoietic Cell Transplantation. ^3^HCT-CI, Hematopoietic Cell Transplantation (HCT)-specific Comorbidity Index. ^4^GVHD, Graft Versus Host Disease. ^5^PTCy, Post-Transplant Cyclophosphamide. ^6^CNI, Calcineurin inhibitor. ^7^MMF, Mycophenolate mofetil. ^8^Sir, Sirolimus.

Values highlighted in bold indicate a statistically significant p value.

At transplant, 220 (80.3%) patients were in first morphological remission and 2 (0.7%) underwent the procedure with active disease. MAC regimens were administered to 145 (53%) patients, and all allo-HCT were performed with PTCY-based prophylaxis. No patient received antithymocyte globulin in the conditioning regimen. Overall, with a median follow-up of 35.5 months, 60 (22.0%) patients experienced disease relapse and 103 (37.6%) patients died. For the entire cohort, the estimated 2-year OS, NRM and CIR rates were 68.5%, 19.4%, and 19.9%, respectively.

### Donor age cut-off determination and impact on outcomes

The median donor age was 38 years (range, 13–75). Overall, 49 (17.9%) donors were female, and in 46 (16.4%) cases, female donors provided grafts to male recipients. All hematopoietic stem cell donations were collected from peripheral blood. In univariate analysis **Table 2A** older donor age (continuous variable) was associated with lower OS (HR: 1.01; 95% CI: 1.01–1.03; p = 0.041) and higher NRM (HR: 1.02; 95% CI: 1.01–1.04; p = 0.009). Importantly, donor age as a continuous variable retained statistical significance in the multivariate analysis for both OS and NRM (HR: 1.034, p = 0.022; HR: 1.04, p = 0.003, respectively).

**Table 2A:** Univariate Cox regression analysis of OS and NRM for the entire cohort.

Univariate analysis	OS^1^ HR^3^ (95% CI^4^)	P value	NRM^2^ HR (95% CI)	P value
Age Continuous Older than 59 years (vs. younger)	1.032 (1.015-1.05) 1.907 (1.273-2.855)	**<0.001** **0.001**	1.037 (1.014-1.061) 1.891 (1.118-3.196)	**0.002** **0.017**
Donor Information Median Age Older than 29 years (vs. younger) Female to Male Stem Cell Products	1.015 (1.001-1.03) 1.638 (1.025-2.615) 0.907 (0.591-1.392)	**0.041** **0.009** 0.274	1.024 (1.006-1.043) 2.056 (1.071-3.946) 1.335 (0.778-2.29)	**0.009** **0.03** 0.31
ELN^5^ Classification III (vs I-II)	1.629 (1.164-2.28)	**0.019**	1.157 (0.796-1.682)	0.7
Disease status: CR^6^2 or more (vs. CR1)	1.042 (0.617-1.759)	0.878	1.342 (0.717-2.512)	0.36
HCT-CI^7^ ≥3 (vs. 0-2)	1.901 (1.094-3.305)	**<0.001**	2.521 (1.259-5.047)	**0.002**
Conditioning Regimen RIC^8^ (vs. MAC^9^)	1.657 (1.11-2.476)	**0.013**	1.569 (0.946-2.602)	0.081
Transplantation period After 2020	1.176 (0.736-1.877)	0.498	0.629 (0.322-1.228)	0.231
Grades 2-4 aGVHD^10^ Time-Dependent Variable	2.303 (0.557-9.519)	0.249	1.402 (0.175-11.23)	0.75
Grades 3-4 aGVHD Time-Dependent Variable	1.858 (1.036-3.333)	**0.037**	2.327 (1.042-5.199)	**0.039**

^1^OS, Overall Survival; ^2^NRM, Non-Relapse Mortality; ^3^HR, Hazard Ratio; ^4^CI, Confidence Interval; ^5^ELN, European LeukemiaNet risk classification. ^6^CR, Complete remission. ^7^HCT-CI, Hematopoietic Cell Transplantation (HCT)-specific Comorbidity Index. ^8^RIC, Reduced Intensity Conditioning; ^9^MAC, Myeloablative Conditioning; ^10^aGVHD, Acute Graft-versus-Host Disease.

Values highlighted in bold indicate a statistically significant p value.

**Table 2B:** Multivariate Cox regression analysis of OS for the entire cohort.

Multivariate analysis	OS^1^ HR^2^ (95% CI^3^)	P value
Age Older than 59 years (vs. younger)	1.02 (1.04-1.04)	**0.016**
Donor Information Older than 29 years (vs. younger)	1.88 (1.11-3.21)	**0.019**
ELN^4^ Classification III (vs I-II)	1.60 (1.05-2.45)	**0.029**
HCT-CI^5^ ≥3 (vs. 0-2)	2.02 (1.35-3.02)	**<0.001**
Conditioning Regimen RIC^6^ (vs. MAC^7^)	0.88 (0.54-1.45)	0.631

^1^OS, Overall Survival; ^2^HR, Hazard Ratio; ^3^CI, Confidence Interval; ^4^ELN, European LeukemiaNet risk classification. ^5^HCT-CI, Hematopoietic Cell Transplantation (HCT)-specific Comorbidity Index. ^6^RIC, Reduced Intensity Conditioning; ^7^MAC, Myeloablative Conditioning.

Values highlighted in bold indicate a statistically significant p value.

**Table 2C:** Multivariate Cox regression analysis of NRM for the entire cohort.

Multivariate analysis	NRM^1^ HR^2^ (95% CI^3^)	P value
Age Older than 59 years (vs. younger)	1.02 (0.99-1.04)	0.09
Donor Information Older than 29 years (vs. younger)	2.03 (1.02-4.13)	**0.049**
HCT-CI^4^ ≥3 (vs. 0-2)	2.06 (1.21-3.49)	**0.007**
Conditioning Regimen RIC^5^ (vs. MAC^6^)	0.89 (0.48-1.67)	0.74

^1^NRM, Non-Relapse Mortality; ^2^HR, Hazard Ratio; ^3^CI, Confidence Interval; ^4^HCT-CI,

Hematopoietic Cell Transplantation (HCT)-specific Comorbidity Index. ^6^RIC, Reduced Intensity Conditioning; ^6^MAC, Myeloablative Conditioning.

Values highlighted in bold indicate a statistically significant p value.

Based on these associations, an optimal donor age cut-off to stratify patients into two risk groups was estimated. Using ROC curve analysis, the optimal donor age cut-off for predicting overall mortality was identified 30 years (AUC = 0.569; 95% CI, 0.502–0.637), and this threshold was used to define the two study cohorts.

### Patient and allo-HCT information according to donor-age groups

As described in **Table 1**, the study cohort was divided into two groups according to donor age showing that 85 (31%) patients received grafts from younger donors, and 189 (69%) from older donors. The median donor age was 24 years (range, 13-30) and 42 years (range, 31-75), respectively. Notably, baseline characteristics and allo-HCT practices were comparable between groups, except for a higher proportion of recipients aged over 65 years in the older donor group (27.5% vs. 11.8%, p = 0.004).

### Post-transplant information and disease relapse according to donor age

The main post-transplant information is reported in **Table 3**. The median time to neutrophil and platelet engraftment was 18 and 24 days, respectively, with no significant differences between patients receiving grafts from younger versus older donors (p = 0.416 and p = 0.948, respectively). Primary or secondary graft failure occurred in 37 (13.9%) patients, with comparable rates between groups (14.5% vs. 13.6%; p = 0.75).

**Table 3:** Main post-transplant information and outcomes.

	All patients N=274	Donor age < 30 years N=85	Donor age ≥ 30 years N=189	P value
Days to neutrophil engraftment, median (IQR)	18 (15-23)	18 (16-21)	18 (15-21)	0.416
Days to platelet engraftment, median (IQR)	24 (16-30)	23 (16-34)	24 (16-29)	0.948
Graft failure, total (%)	37 (13.9)	12 (14.5)	25 (13.6)	0.75
SOS^1^, total (%)	11 (4)	5 (5.9)	6 (3.2)	0.326
TA-TMA^2^, total (%)	17 (6.3)	4 (4.8)	13 (6.9)	0.596
Infectious Complications During First 180 Days				
Bacteraemia, total (%)	122 (44.5)	43 (50.6)	79 (42.2)	0.237
CMV^3^ reactivation, total (%)	168 (63.6)	41 (60.3)	127 (64.8)	1
CMV disease, total (%)	23 (8.7)	7 (10.3)	16 (8.2)	0.523
PTLD^4^, total (%)	0 (0)	0 (0)	0 (0)	1
Cumulative Incidences of GVHD % (95% CI)				
Day 100 Grades II-IV aGVHD	15.6 (11.5-20.2)	3.0 (0.6-9.3)	19.9 (14.6-25.8)	**<0.001**
Day 100 Grades III-IV aGVHD	8.0 (5.1-11.7)	1.5 (0.1-7.2)	10.2 (6.5-14.9)	**0.034**
2-Y Mod/Sev cGVHD	11.5 (7.7-16.0)	11.7 (5.1-21.3)	11.4 (7.1-16.7)	0.49
Relapse, total (%)	60 (21.9)	17 (20)	43 (22.8)	0.64
Dead, total (%)	103 (37.6)	24 (28.2)	79 (42)	**0.032**
Directly secondary to NRM^5^, total (%)	57 (20.8)	11 (12.9)	46 (24.3)	**0.016**
Main Causes of Death (CIBMTR)				
Relapse	40 (38.8)	5 (20.8)	35 (44.3)	0.055
Infections	32 (31.1)	8 (33.3)	24 (30.4)	0.805
GVHD	10 (9.7)	1 (4.2)	9 (11.4)	0.446
Graft Failure	4 (3.9)	0 (0)	4 (5.1)	0.571
Multi-organ failure	2 (1.9)	1 (4.2)	1 (1.3)	1
Others	15 (14.6)	9 (37.5)	6 (7.6)	**0.001**
Main Post-Transplant Outcomes [% (95% CI)]				
2-y Overall Survival	68.5 (62.5-73.8)	80.6 (68.9-88.2)	64.3 (57.0-70.7)	**0.011**
2-y Relapse-Free Survival	60.7 (54.4-66.3)	73.1 (60.7-82.1)	56.3 (48.9-63.0)	**0.012**
2-y Non-Relapse Mortality	19.4 (14.9-24.4)	11.1 (5.4-19.1)	23.2 (17.3-29.5)	**0.031**
2-y Cumulative Incidence of Relapse	19.9 (15.2-25.0)	18.6 (11.0-27.8)	20.6 (14.9-26.9)	0.599
2-y GRFS^6^	0.51 (0.45-0.57)	0.61 (0.49-0.72)	0.48 (0.41-0.55)	0.067

^1^SOS: Sinusoidal obstruction syndrome. ^2^TMA: Thrombotic Microangiopathy. ^3^CMV: cytomegalovirus. 4PTLD: Post-Transplant Lymphoproliferative Syndrome. ^5^NRM: Non-Relapse Mortality. ^6^GRFS: Graft- versus-host disease-free, Relapse-free survival.

Values highlighted in bold indicate a statistically significant p value.

The incidence of veno-occlusive disease and post-transplant thrombotic microangiopathy was 5.9% and 4.8%, respectively, among recipients receiving grafts from younger donors, and 3.2% (p = 0.326) and 6.9% (p = 0.596) among those receiving grafts from older donors. Infectious complications during the first 180 days post-transplant were frequent regardless of donor age, with similar rates of bloodstream infections (50.6% vs. 42.2%; p = 0.237), CMV reactivation (60.3% vs. 64.8%; p = 1), and CMV disease (10.3% vs. 8.2%; p = 0.523) in both groups.

**Figure 1:**
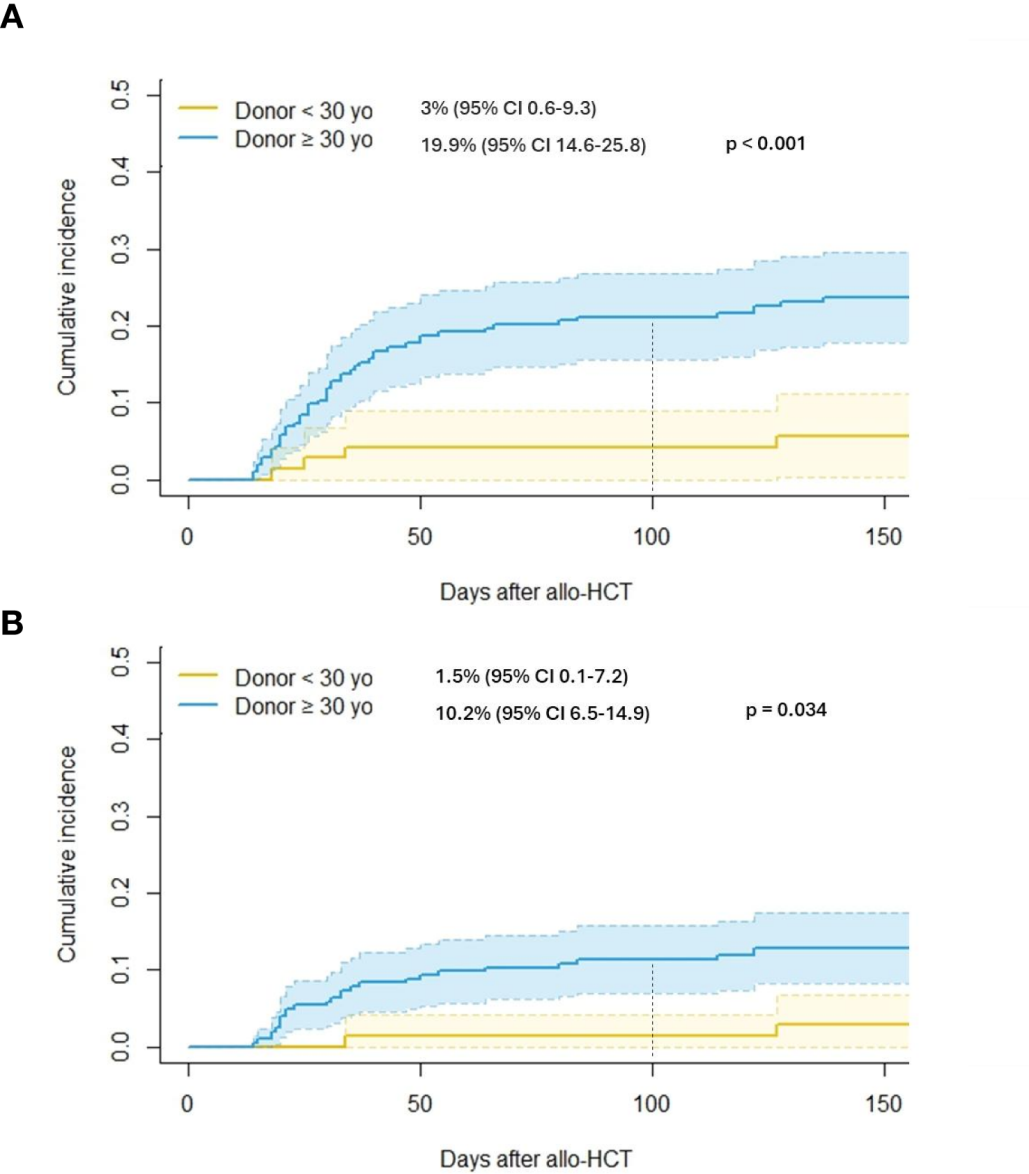
**(A)**. Cumulative incidence of grade II-IV aGVHD according to donor age. **(B)** Cumulative incidence of grade III-IV aGVHD according to donor age.

Notably, patients who received grafts from younger donors had a significantly lower incidence of aGVHD, both for grade II–IV (Day +100: 3.0% vs. 19.9%; p < 0.001; **Figure 1A**) and for severe grade III–IV (Day +100: 1.5% vs. 10.2%; p = 0.034; **Figure 1B**). However, the incidence of moderate-to-severe cGVHD at two years was similar between groups (11.7% vs. 11.4%; p = 0.49). Donor age—particularly when over 50 years (OR 4.9, p = 0.016)—and female recipient (OR 3.8, p = 0.045) were independently associated with a significantly increased risk of developing GVHD. This association remained statistically significant in the multivariate analysis (**Tables 4A**, **B**).

Disease relapse occurred in 17 (20.0%) patients transplanted from younger donors and in 43 (22.8%) patients from donors aged 30 years or older. The two-year cumulative incidence of relapse was 18.6% and 20.6%, respectively (p = 0.599).

**Table 4A:** Univariate logistic regression analysis to predict acute graft-versus-host disease.

Variables	Univariate analysis			
	OR^1^	Inferior 95% CI^2^	Superior 95% CI	*p* value
Patient age Older than 59 years	1.03 2.24	0.97 0.56	1.10 8.98	0.320 0.255
Donor age Older than 49 years	1.05 4.93	1.01 1.35	1.11 18.0	**0.048** **0.016**
RIC^3^ (vs. MAC^4^)	0.61	0.17	2.15	0.446
Female recipient	3.80	1.03	14.0	**0.045**
Female donor	1.22	0.33	4.53	0.761
HCT-CI^5^	2.05	0.32	13.3	0.454

^1^OR: Odds ratio; ^2^CI: Confidence Interval; ^3^RIC: Reduced Intensity Conditioning; ^4^MAC: Myeloablative Conditioning; ^5^HCT-CI: Hematopoietic Cell Transplantation (HCT)-specific Comorbidity Index.

Values highlighted in bold indicate a statistically significant p value.

**Table 4B:** Multivariate logistic regression analysis to predict acute graft-versus-host disease.

Variables	Multivariate analysis			
	OR	Inferior 95% CI	Superior 95% CI	*p* value
Donor age Older than 59 years	4.55	1.20	17.3	**0.026**
Female recipient	3.94	0.99	15.5	0.050

Values highlighted in bold indicate a statistically significant p value.

### Donor age and post-transplant outcomes

As shown in **Table 3**, mortality rate was significantly lower in patients receiving grafts from donors younger than 30 years compared to those in the older donor group (28.2% vs. 42.0%, p = 0.032), as interestingly, main causes of death were attributable to NRM (12.9% vs. 24.3%, p = 0.016). Regarding the causes of death, they were comparable between groups, with infections representing the leading cause in both cohorts (33.3% vs. 30.4%, P = 0.805). Miscellaneous causes (hemorrhage, stroke, arrhythmia, respiratory failure, thrombotic microangiopathy and sinusoidal obstruction syndrome) accounted for 37.5% of deaths in the younger donor group, while GVHD represented 11.4% of deaths in the older donor group.

**Figure 2:**
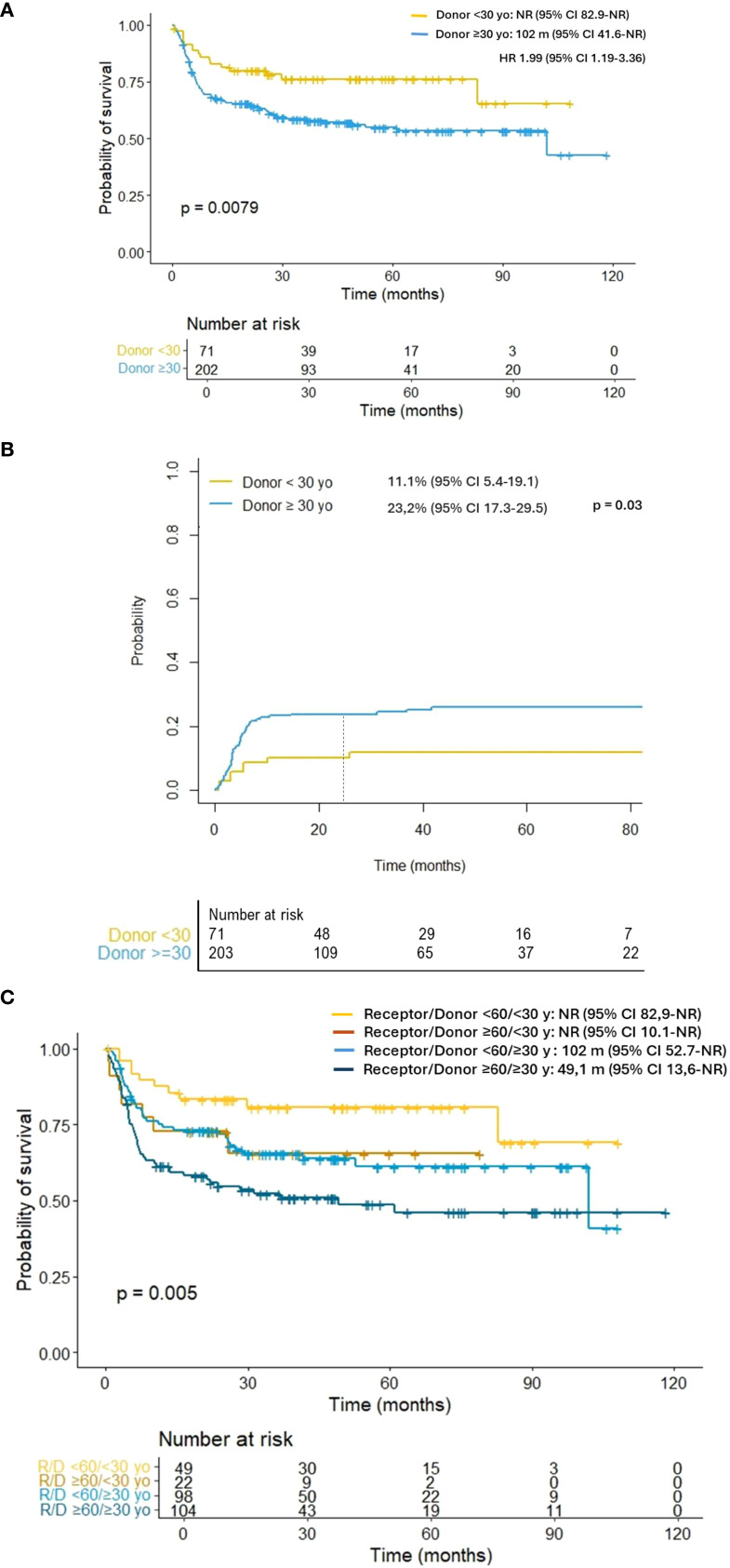
**(A)** Overall survival according to donor age. **(B)** Non-relapse mortality according to donor age. **(C)** Overall survival according to donor and receptor age. Individuals over 60 years who received a transplant from a younger donor demonstrated overall survival comparable to that of patients under 60 years who received grafts from older donors (101 months vs NR; p = 0.889).

As illustrated in **Figure 2A**, patients receiving grafts from younger donors had higher OS (2- year: 80.6% vs. 64.3%, p = 0.011) and lower NRM (11.1% vs. 23.2%, p = 0.031; **Figure 2B**) than those included in the older donor cohort. Regarding patient age, individuals over 60 years who received a transplant from a younger donor demonstrated OS comparable to that of patients under 60 years who received grafts from older donors (102 months vs NR; p = 0.889; **Figure 2C**). In addition, younger donor cohort exhibited a significantly higher RFS (2-year: 73.1% vs. 56.3%, p = 0.012) due to the reduced rates of NRM observed in this group of patients.

### Donor age as an independent predictive factor for survival

As shown in **Table 2A**, the UVA indicated that patients undergoing haplo-HCT from donors aged ≥30 years had significantly worse OS (HR: 1.63; 95% CI: 1.02–2.61; p = 0.009) and higher NRM risk (HR: 2.05; 95% CI: 1.07–3.94; p = 0.03). Based on these findings, a MVA was performed, including the variables found to be significant in the UVA and other considered clinically relevant. As shown in **Table 2B**, the MVA confirmed that grafts from older donors were associated with worse OS (HR: 1.88; 95% CI: 1.10–3.20; p = 0.019) and higher NRM (HR: 2.06; 95% CI: 1.01–4.13; p = 0.049) (**Table 2C**).

Additionally, the MVA for OS revealed that patients older than 59 years (HR 1.02, p = 0.016), those with high-risk AML (HR: 1.60; p = 0.029), and those with an HCT-CI score >3 (HR: 2.01; p < 0.001) had an increased risk of mortality. For NRM, a higher comorbidity burden (HCT-CI >3; HR: 2.06; p = 0.007) and age over 59 years (HR: 1.02; p = 0.09) were also identified as additional risk factors.

## Discussion

Haplo-HCT with PTCY-based prophylaxis represents a safe and effective strategy that has expanded access to transplantation for patients without a suitable HLA-matched donor. However, optimal criteria for donor selection remain under investigation. In this multicenter, retrospective analysis, we evaluated 274 patients who underwent haplo-HCT. Our findings indicate that donor age is an independent and significant factor, with younger donor age than 30 years associated with improved OS, reduced NRM and a lower incidence of clinically relevant aGVHD.

Donor age has emerged one of the main factors investigated in the search for optimal donor characteristics to improve allo-HCT outcomes in the recent years. Previous evidence conducted in HLA-matched allo-HCT settings has demonstrated the significant role of younger donor age in outcomes (5). However, older sibling donors are generally linked to older patients limiting the evaluation of the independent effects of both variables on transplant results. Notably, recent studies in the context of MUD allo-HCT have further demonstrated that selecting younger donors increases the likelihood of transplantation success. Specifically, younger donor age has been associated with lower incidences of GVHD, improved immune reconstitution, and superior overall survival, thereby underscoring the critical impact of donor age on transplantation outcomes (4, 6, 7).

Over the past 15 years, the indication of haplo-HCT has steadily increased and currently represents approximately 20% of all allogeneic HCTs performed in Europe (8, 9). This expansion is largely attributable to the adoption of PTCy-based prophylaxis, which has revolutionized the field of allo-HCT due to its immunomodulatory effects for GVHD prevention and favorable safety profile (10). Consistently, many patients who previously lacked a suitable HLA-matched donor are now able to safely undergo haplo-HCT. In addition, among haploidentical donors, the degree of relatedness can vary—typically including children, siblings, and parents—leading to a wide range of donor ages within this group, justifying the importance of refining donor selection criteria in the haplo-HCT setting and determining the role of donor age in haplo-HCT outcomes. In AML, where relapse risk remains a major barrier to cure, the more widespread use of PTCy platforms not only increases donor availability but also shifts the focus toward donor optimization. Recent large-scale analyses in the PTCy era, reported by Sanz J. et al. and Ye Y. et al. (11, 12), indicate that donor-related factors such as age and relationship now emerge as independent predictors of outcome, suggesting that donor selection—beyond mere donor availability—is increasingly relevant for AML patients undergoing haplo-HCT.

According to our results, the infusion of peripheral blood stem cells (PBSC) products from haploidentical donors younger than 30 years was associated with higher OS, lower NRM and comparable relapse rates. These results are particularly relevant in haplo-HCT settings, where potential donors often span a wide age range, including siblings, parents, children, and other relatives. As illustrated in **Figure 2C**, older patients (≥60 years) who received grafts from donors under 30 years exhibited survival outcomes comparable to younger recipients transplanted from older donors (101 months vs. not reached; p = 0.889). This 30-year cut-off should be interpreted with caution due to the limited statistical power, and further research is needed to refine the optimal threshold. Nevertheless, it provides a clinically applicable criterion for donor selection.

Our results are consistent with previous data reported by DeZern et al. (13), further supporting the favorable impact of selecting younger donors in the haplo-HCT setting performed with non-myeloablative conditioning regimens, and support transplant practices at different institutions were a youngest adult-sized haploidentical donor is selected if feasible and medically appropriate. Moreover, conclusions are particularly relevant for AML patients, a disease frequently affecting elderly patients who often have older siblings as potential donors (14).

Notably, our results also demonstrate that patients receiving grafts from younger donors exhibited a significantly lower incidence of aGVHD, both grade II–IV and grade III–IV (3.0% vs. 19.9%, p < 0.001; and 1.5% vs. 10.2%, p = 0.034, respectively). In contrast, no significant differences were observed in the incidence of cGVHD (11.7% vs. 11.4%; p = 0.49). These findings are clinically relevant, given that GVHD remains a major source of morbidity and mortality following allo-HCT, and its prevention is a central therapeutic objective, particularly in the haploidentical setting. In addition, observations are consistent with prior reports (7, 13), where younger donor age has been associated with lower aGVHD rates and improved immune reconstitution. It is hypothesized that younger donors provide a higher proportion of functional, naïve T cells with distinct cytokine profiles, contributing to reduced alloreactivity and GVHD risk.

In contrast to the significant differences in aGVHD, our study found no differences in the relapse incidence between younger and older donor cohorts (18.6% vs. 20.6%, p = 0.599). This finding is consistent with previous studies suggesting that donor age exerts limited impact on graft-versus-leukemia effects (7, 13). Thus, the survival benefit observed with younger donors appears to be primarily driven by reduced NRM, rather than differences in disease control. Overall, these results reinforce the notion that relapse risk is determined by a complex interplay of factors beyond donor age, including leukemia biology, conditioning regimen intensity, and post-transplant immunosuppressive strategies (15). In this context, selecting younger donors may help minimize transplant-related complications without compromising anti-leukemic efficacy.

Grafts obtained from younger donors may possess more favorable biological characteristics compared to those from older donors. Aged HSCs exhibit impaired self-renewal and long-term reconstitution potential (16). Moreover, clonal hematopoiesis increases with age, with a prevalence of approximately 1% in healthy individuals under 40 years and up to 20% in those over 65 and has been associated with shorter OS and a higher risk of relapse (17). HCT from younger donors are associated with improved immune reconstitution, based on faster kinetics of CD4+ T cell, CD8+ T cell, B cell and NK cell development (18). Age-related differences in lymphoid cell populations may also influence haplo-HCT outcomes. In this context, the presence of innate lymphoid cells in allogeneic grafts has been reported to reduce the incidence of GVHD (19), potentially reflecting impaired alloreactivity in grafts from older donors. Nevertheless, the underlying causes of the improved outcomes observed with younger donors have not yet been well characterized. In this regard, further research is needed to clarify the interplay between biological and clinical factors that may explain the findings observed in the present study.

Our study has several limitations. The retrospective design inherently carries a risk of selection and reporting biases, and the restriction to centers within a single country and a specific disease may limit the generalizability of our findings to broader or more diverse populations. In addition, the present analysis focuses its investigation on donor age and does not include information about donor/recipient CMV serostatus or ABO compatibility. Although the results are of interest, caution is needed in their interpretation, as other relevant donor-related variables were not included in this study. To further address this limitation, future analyses will be conducted to explore their potential impact on transplant outcomes. In the present study, CMV serostatus was not included at the time of data collection since part of the patients transplanted after 2021 received letermovir prophylaxis. Moreover, the impact of ABO compatibility on haplo-HCT outcomes has already been investigated in previous reports, supporting the consideration of this variable as well when selecting haploidentical donors for transplantation (20).

In summary, our findings confirm that donor age is an independent determinant of outcomes in haplo-HCT, with younger donors associated with improved OS, reduced NRM, and lower incidence of aGVHD. This effect remains significant regardless of recipient age and appears unrelated to relapse risk. The results underscore the importance of donor age as a key consideration in donor selection in AML patients. Biological factors such as clonal hematopoiesis and immune cell composition may underline these associations.

## Data Availability

The raw data supporting the conclusions of this article will be made available by the authors, without undue reservation.
